# Absence of γ-sarcoglycan alters the response of p70S6 kinase to mechanical perturbation in murine skeletal muscle

**DOI:** 10.1186/2044-5040-4-13

**Published:** 2014-07-01

**Authors:** Catherine Moorwood, Anastassios Philippou, Janelle Spinazzola, Benjamin Keyser, Edward J Macarak, Elisabeth R Barton

**Affiliations:** 1 Department of Anatomy and Cell Biology, School of Dental Medicine, University of Pennsylvania, Philadelphia, PA, USA; 2 Pennsylvania Muscle Institute, University of Pennsylvania, Philadelphia, PA, USA; 3 Current address: Department of Physiology, Medical School, National and Kapodistrian University of Athens, Goudi, Athens, Greece; 4 Current address: Department of Dermatology and Cutaneous Biology, Jefferson University College of Medicine, Philadelphia, PA, USA

**Keywords:** Sarcoglycan, Sarcoglycanopathies, Limb girdle muscular dystrophy, Mechanotransduction, Mechano-sensing, Load-sensing, p70S6K, S6K, p70S6 kinase, ERK1/2

## Abstract

**Background:**

The dystrophin glycoprotein complex (DGC) is located at the sarcolemma of muscle fibers, providing structural integrity. Mutations in and loss of DGC proteins cause a spectrum of muscular dystrophies. When only the sarcoglycan subcomplex is absent, muscles display severe myofiber degeneration, but little susceptibility to contractile damage, suggesting that disease occurs not by structural deficits but through aberrant signaling, namely, loss of normal mechanotransduction signaling through the sarcoglycan complex. We extended our previous studies on mechanosensitive, γ-sarcoglycan-dependent ERK1/2 phosphorylation, to determine whether additional pathways are altered with the loss of γ-sarcoglycan.

**Methods:**

We examined mechanotransduction in the presence and absence of γ-sarcoglycan, using C2C12 myotubes, and primary cultures and isolated muscles from C57Bl/6 (C57) and γ-sarcoglycan-null (γ-SG^-/-^) mice. All were subjected to cyclic passive stretch. Signaling protein phosphorylation was determined by immunoblotting of lysates from stretched and non-stretched samples. Calcium dependence was assessed by maintaining muscles in calcium-free or tetracaine-supplemented Ringer’s solution. Dependence on mTOR was determined by stretching isolated muscles in the presence or absence of rapamycin.

**Results:**

C2C12 myotube stretch caused a robust increase in P-p70S6K, but decreased P-FAK and P-ERK2. Neither Akt nor ERK1 were responsive to passive stretch. Similar but non-significant trends were observed in C57 primary cultures in response to stretch, and γ-SG^-/-^ cultures displayed no p70S6K response. In contrast, in isolated muscles, p70S6K was mechanically responsive. Basal p70S6K activation was elevated in muscles of γ-SG^-/-^ mice, in a calcium-independent manner. p70S6K activation increased with stretch in both C57 and γ-SG^-/-^ isolated muscles, and was sustained in γ-SG^-/-^ muscles, unlike the transient response in C57 muscles. Rapamycin treatment blocked all of p70S6K activation in stretched C57 muscles, and reduced downstream S6RP phosphorylation. However, even though rapamycin treatment decreased p70S6K activation in stretched γ-SG^-/-^ muscles, S6RP phosphorylation remained elevated.

**Conclusions:**

p70S6K is an important component of γ-sarcoglycan-dependent mechanotransduction in skeletal muscle. Our results suggest that loss of γ-sarcoglycan uncouples the response of p70S6K to stretch and implies that γ-sarcoglycan is important for inactivation of this pathway. Overall, we assert that altered load-sensing mechanisms exist in muscular dystrophies where the sarcoglycans are absent.

## Background

The dystrophin glycoprotein complex (DGC) is found at the sarcolemma of skeletal, cardiac, and smooth muscle cells, where it physically links the extracellular matrix (ECM) with the intracellular cytoskeleton, providing structural support [1-3]. Mutations in DGC components cause different types of muscular dystrophy; for example, mutations in dystrophin cause Duchenne muscular dystrophy (DMD), while mutations in α-, β-, γ-, or δ-sarcoglycan (SG) cause limb girdle muscular dystrophy (LGMD) [2-4]. When dystrophin is mutated in DMD or in the *mdx* mouse model of DMD, the entire DGC is substantially reduced at the sarcolemma. In contrast, when any one of the SGs is mutated, in LGMD or any of the SG knock-out mice, the other three SGs are either absent or reduced at the sarcolemma, but the rest of the DGC remains, including the link formed by dystrophin and dystroglycan between the ECM and the cytoskeleton [2,3,5,6]. Unlike the skeletal muscles of the *mdx* mouse, muscles of the γ-SG knock-out (γ-SG^-/-^) mouse display no mechanical fragility, at least until 4 months of age, as shown by a minimal loss of force-generating capacity following a series of eccentric contractions (ECCs) [7,8]. In spite of this, the γ-SG^-/-^ mouse exhibits a severe dystrophic phenotype on histological examination, with extensive myofiber degeneration and regeneration, fibrosis, and disruption of sarcolemmal integrity, similar to the *mdx* mouse [9]. The lack of mechanical fragility suggests that aberrant signaling may contribute to the muscle degeneration seen in the γ-SG^-/-^ mouse. Indeed, our previous studies demonstrated that localization of the SG complex to the sarcolemma and phosphorylation of the tyrosine 6 residue of γ-SG are essential for normal signaling by extracellular signal-regulated kinases 1 and 2 (ERK1/2), in response to ECCs [10,11]. Based upon these data, we have asserted that the SG complex acts as a mechanosensor in skeletal muscle because of its position within the DGC, the modifications that occur to γ-SG with mechanical perturbation, and the necessity of the complex for normal signaling.

A complication of using ECCs to invoke signal transduction is that there are multiple processes in play. Not only is there externally applied tension on the sarcolemma and DGC as a result of lengthening, but also active contraction resulting in internally applied tension and Ca^2+^ flux within the fibers and across the sarcolemma, all of which could contribute to alterations in signaling in the absence of γ-SG. Calcium is known to be involved in many mechanosensitive signaling pathways (reviewed in [12,13]) and aberrant calcium regulation is a feature of SG-deficient muscle [14-22]. Indeed, an exaggerated ERK1/2 response to mechanical stimulation that is dependent on extracellular calcium has been demonstrated in the *mdx* mouse diaphragm [23]. Studies suggest that aberrant calcium regulation in the muscular dystrophies results from abnormal levels and activity of the mechanosensitive TRP channels and/or misregulation of store operated calcium entry via the STIM1 and Orai1 channels [24-29]. Direct disruption of the sarcolemma, for which there is evidence in SG-null animal models [7,9,30,31], could also contribute to loss of calcium homeostasis. Furthermore, several strategies to improve Ca^2+^ handling are known to counteract the pathology associated with the muscular dystrophies [25,32-34]. Therefore, identification of mechanosensitive signaling that is attributable to the SG complex rather than other processes occurring during mechanical perturbation has been challenging.

One pathway of interest involves p70S6K, which is canonically activated in response to mitogens via the phosphoinositide 3-kinase (PI3K) pathway (reviewed in [35]) and is known to respond to mechanical load [36]. Activation of p70S6K involves a hierarchical series of phosphorylation events, beginning with phosphorylation of multiple sites in the C-terminal autoinhibitory domain, followed by mammalian target of rapamycin (mTOR)-dependent phosphorylation of sites in the linker region, which allows for full activation of the kinase via phosphorylation of threonine 229 (T229) in the catalytic domain by phosphoinositide-dependent kinase 1 (PDK1) (reviewed in [37]). Although phosphorylation of T229 is required for p70S6K activation, phosphorylation of T389 in the linker region has been found to correlate most closely with *in vivo* activity [38], and can be used as a measure of kinase activation. p70S6K has a multitude of downstream targets, with roles in protein synthesis, growth, proliferation, survival, and more [35], including S6 ribosomal protein (S6RP), which closely correlates with protein translation rates [39].

In the current study, we examined ERK1/2, Akt, focal adhesion kinase (FAK), and p70S6K responses to passive stretch in C57 and γ-SG^-/-^ skeletal muscle to further elucidate the importance of the SG complex for mechanotransduction. While differences in ERK1/2 phosphorylation between C57 and γ-SG^-/-^ muscle were calcium-dependent, differences in p70S6K activation were independent of calcium. In addition, the p70S6K response to stretch in both primary myofiber cultures and isolated extensor digitorum longus (EDL) muscles was differentially regulated in the absence of γ-SG. Specifically, experiments in isolated muscles suggest that γ-SG is required for inactivation of p70S6K. The findings increase our understanding of the contribution of aberrant load-sensing to the pathology of muscular dystrophies where the SG complex is deficient.

## Methods

### Animals

Adult C57BL/6 (C57) and γ-SG-null (γ-SG^-/-^) mice were used. For *ex vivo* protocols, mice were aged 8 to 16 weeks. The γ-SG^-/-^ mouse lacks γ-SG due to gene targeting, resulting in an additional loss of β- and δ-SG and a decrease of α-SG [9]. All experiments were approved by the University of Pennsylvania Institutional Animal Care and Use Committee.

### C2C12 myotube culture

Flexible silicone membranes (Specialty Manufacturing, Inc.) were stretched across the bottom of custom cylinders which acted as a culture chamber. The membranes were held in place using an O-ring as described previously [40]. Membranes were then coated with a thin layer of 2 mg/mL GFR Matrigel (BD #354230). C2C12 myoblasts (3.5 × 10^5^/cylinder) were seeded onto the membranes and maintained at 5% CO_2_ at 37°C in growth media (10% FBS, 100 U penicillin, 100 μg streptomycin, 100 μg/mL gentamycin in DMEM) for approximately 24 h until 70% to 80% confluent, then switched to differentiation media (2% HS, 100 U penicillin, 100 μg streptomycin, 100 μg/mL Gentamycin in DMEM). Myoblasts were allowed to differentiate into multinucleated myotubes for 5 days, during which media was changed every other day before stretching as described below.

### Primary myoblast culture

Mice were euthanized using CO_2_ inhalation. Flexor digitorum brevis (FDB) muscles were dissected and incubated with 2 mg/mL collagenase I (Sigma), 10% FBS in Tyrode’s solution (125 mM NaCl, 5 mM KCl, 1 mM CaCl_2_, 1 mM MgCl_2_, 1 mM KH_2_PO_4_, 20 mM HEPES (all from Fisher), 5.5 mM glucose (Sigma), pH 7.4) for 90 min at 37°C, with shaking, as previously described [41]. Muscles were washed in 10% FBS in Tyrode’s solution and clumps of fibers were liberated by pipetting up and down in 10% FBS, 100 U penicillin, 100 μg streptomycin in Tyrode’s solution using a wide-mouthed glass pipette. Clumps of fibers were transferred to a second dish with the same solution and pipetted up and down again. Single fibers were transferred to a third dish before plating on silicone membranes coated with Matrigel (Becton Dickinson; 2 mg/mL diluted in DMEM). Growth media (20% FBS, 10 ng/mL mouse basic fibroblast growth factor (MP Bio), 100 U penicillin, 100 μg streptomycin, 1 μg/mL Fungizone, 100 μg/mL Gentamycin in Ham’s F-10 media) was carefully added and cultures were incubated without disturbance for 3 days at 37°C. Media was then changed every 2 days. After 7 to 10 days, when myoblast cultures were 70% to 80% confluent, media was switched to differentiation media (10% HS, 0.5% chicken embryo extract, 100 U penicillin, 100 μg streptomycin, 1 μg/mL Fungizone, 100 μg/mL Gentamycin in DMEM) and myoblasts were allowed to differentiate into multinucleated myotubes for 5 days before stretching as described below. Unless otherwise indicated, all cell culture reagents were purchased from Gibco.

### Myotube stretching protocol

C2C12 myotubes were stretched using an apparatus that produces isotropic two-dimensional strain of cells *in vitro*, as described previously [40]. Briefly, myotubes were subjected to 10% strain, 40 times per minute, for 30 min, at 37°C in a humidified atmosphere of 5% CO_2_ in air. Control myotubes were treated identically but were not stretched. Lysates were harvested immediately as described below. Primary myotubes were stretched using the protocol described for C2C12 myotubes. Stretched myotubes were then incubated without stretch for a further 1, 2, or 4 h at 37°C before harvesting lysates for immunoblotting as described below. Control (non-stretched) myotubes were harvested immediately after the end of the stretching protocol used for the stretched myotubes.

### Isolated muscle stretching protocol

Mice were anaesthetized using ketamine and xylazine. EDL muscles were dissected and placed in an organ bath containing oxygenated high-glucose (25 mM) DMEM with HEPES (25 mM) (Life Technologies), at room temperature. For rapamycin sensitivity experiments, high-glucose DMEM was supplemented with 150 nM rapamycin (Sigma) or vehicle only (0.1% DMSO). Muscles were adjusted to 9.3 mN of resting tension, approximately equivalent to optimal length, based on our previous experiments [42]. After a 10-min equilibration period, the length of the muscle was measured using calipers, and the muscle was subjected to a stretching protocol of 15% strain (held for 100 ms with ramp times of 50 ms), 20 times per minute, for either 30 or 90 min, using an *in vitro* muscle test system (Aurora Scientific). Muscles were snap-frozen immediately following the end of the stretch protocol. Contralateral muscles were used as controls and were adjusted to the same length as the stretched muscles, then incubated in oxygenated high-glucose DMEM with HEPES at room temperature for the equivalent length of time, before snap-freezing.

### Calcium dependence experiments

EDL muscles were dissected as described above and placed in an organ bath contained oxygenated Ringer’s solution (119 mM NaCl, 4.74 mM KCl, 2.54 mM CaCl_2_, 1.18 mM KH_2_PO_4_, 1.18 mM MgSO_4_, 25 mM HEPES, 2.75 mM glucose), calcium-free Ringer’s solution (CaCl_2_ replaced with 2.5 mM MgCl_2_) or Ringer’s solution supplemented with 100 μM tetracaine. They were incubated for 30 min before snap-freezing in liquid nitrogen.

### Immunoprecipitation

Immunoprecipitation experiments were carried out using the Pierce Classic IP kit (Thermo Scientific). Muscle lysates containing 100 μg total protein was immunoprecipitated with anti-P-tyrosine (Cell Signaling #9411 1:100) overnight at 4°C with end-over-end mixing. Samples were purified using Protein A/G Plus Agarose beads (Roche) for 1 h at 4°C with end-over-end mixing. The immune complex was eluted with non-reducing sample buffer and boiled at 100°C for 5 min before being applied to a SDS-PAGE gel, transferred, and immunoblotted as described below.

### Immunoblotting

C2C12 and primary myotubes were washed with ice-cold PBS before lysing in 100 to 200 μL of RIPA buffer (50 mM HEPES pH 7.5, 150 mM NaCl, 5 mM EDTA, 1 mM EGTA, 15 mM p-nitrophenyl phosphate disodium hexahydral, 1% NP-40, 0.1% SDS, 1% deoxycholate, 0.025% sodium azide) with protease and phosphatase inhibitor cocktails (Sigma). Lysates were incubated on ice for 30 min, centrifuged at 16,000 rcf for 20 min at 4°C and the supernatants retained. EDL muscles were ground using a dry ice-cooled pestle and mortar, and lysed in 200 μL of RIPA buffer with protease and phosphatase inhibitors. Lysates were incubated on ice for 1 h, vortexing half-way through, centrifuged at 16,000 rcf for 20 min at 4°C and the supernatants retained. Protein content was determined using a Bradford method protein assay kit (Bio-Rad). Lysates (30 μg total protein for myotube cultures, 90 μg total protein for EDL muscles) were separated by SDS-PAGE on Tris-HCl polyacrylamide gels (Bio-Rad) and transferred to PVDF membranes. Membranes were blocked in 5% milk in TTBS (10 mM Tris, 150 mM NaCl, 0.1% v/v Tween-20, pH 8), with 2% BSA added for some antibodies, then probed with antibodies to the following: phospho (P)-p70S6K (T389) (Cell Signaling #9205 1:200 for primary cultures, Cell Signaling #9234 1:250 for isolated muscles), P-p70S6K (T421/S424) (Cell Signaling #9204 1:1,000), P-S6RP (Cell Signaling #2211 1:2,000), P-ERK1/2 (Cell Signaling #9101 1:500), total (T)-ERK1/2 (Cell Signaling #9107 1:1,000), P-Akt (Cell Signaling #9271 1:300), T-Akt (Cell Signaling #2920 1:2,000), P-FAK (Millipore 05-1140, 1:500), T-FAK (Millipore 06-543, 1:1,000), γ-SG (Novocastra VP-G803 1:300), glyceraldehyde 3-phosphate dehydrogenase (GAPDH) (Santa Cruz sc-32233 1:5,000), and tubulin (Sigma T5168 1:20,000). Band intensities were quantified using ImageQuant TL, 1D gel analysis (C2C12 and myotube cultures, rapamycin sensitivity experiments) or ImageJ (NIH) (all other isolated muscles). P-p70S6K and P-S6RP were normalized to either GAPDH or tubulin, P-FAK was normalized to either T-FAK or GAPDH, P-Akt was normalized to T-Akt, and P-ERK1/2 was normalized to T-ERK1/2.

### Microscopy

Images of primary cultures were taken using a Leica DM RBE microscope and Leica DFC300 CCD camera, using OpenLab software (Perkin Elmer).

### Statistics

**Figure 1:**
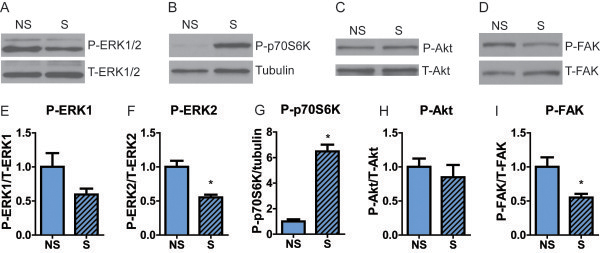
**p70S6K responds to stretch in C2C12 cells.** C2C12 myotubes were cultured on silicone membranes and subjected to passive stretching for 30 min. **(A-D)** Representative immunoblots for P-ERK1/2, total T-ERK1/2, P-p70S6K (T389 site), tubulin, P-Akt, T-Akt, P-FAK, and T-FAK in non-stretched (NS) and stretched (S) C2C12 cells. **(E-I)** Quantification of activation levels. P-ERK1 and 2 were normalized to T-ERK1 and 2, respectively, P-p70S6K was normalized to tubulin, P-Akt was normalized to T-Akt, and P-FAK was normalized to T-FAK. n = 5-6 wells of C2C12 cells per group. Bars represent mean ± standard error. * Significantly different from non-stretched myotubes by unpaired T test. NS, non-stretched; S, stretched.

**Figure 2:**
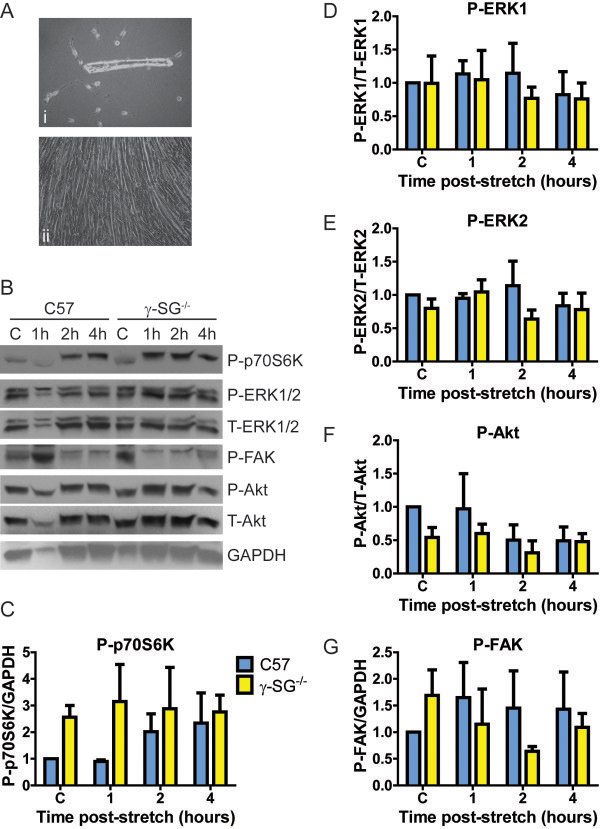
**Differential p70S6K stretch response in C57 and γ-SG**^**-/- **^**primary cultures.** Primary myotubes from C57 and γ-SG^-/-^ FDB fibers were cultured on silicone membranes and subjected to passive stretching for 30 min. Lysates were harvested 1, 2, or 4 h after stretch. **(A)** Representative images of i, satellite cells migrating from an FDB fiber and ii, differentiated myotubes. **(B)** Representative immunoblot for P-p70S6K (T389 site), P- and T-ERK1/2, P-FAK, P-Akt, T-Akt, and GAPDH in non-stretched (C) and stretched (1 h, 2 h, 4 h) primary cultures. **(C-G)** Quantification of activation levels. Legend in C applies to all graphs. P-ERK1 and 2 were normalized to T-ERK1 and 2, respectively, P-Akt was normalized to T-Akt and P-p70S6K and P-FAK were normalized to GAPDH. n = 3 (p70S6K) or 4 (all other proteins) independent sets of primary cultures per genotype. Bars represent mean ± standard error. Statistical significance was tested by two-way ANOVA.

**Figure 3:**
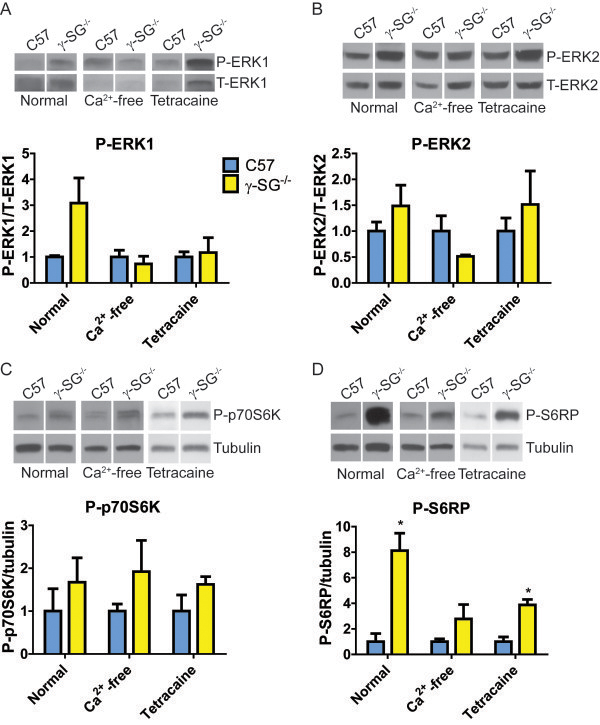
**Elevated p70S6K in γ-SG**^**-/- **^**muscles is independent of calcium.** EDL muscles from C57 and γ-SG^-/-^ mice were maintained in normal oxygenated Ringer’s solution, calcium-free oxygenated Ringer’s solution or oxygenated Ringer’s solution supplemented with tetracaine for 30 min. **(A-D)** Representative immunoblots and quantification for P-ERK1 **(A)**, P-ERK2 **(B)**, P-p70S6K (T389 site; **C**), and P-S6RP **(D)**. Legend in A applies to all graphs. P-ERK 1 and 2 were normalized to T-ERK 1 and 2, respectively; P-p70S6K and P-S6RP were normalized to tubulin. Independent immunoblots were performed for each condition and γ-SG^-/-^ activation levels were normalized to C57 activation levels in each case. n = 3 muscles per genotype and condition. Bars represent mean ± standard error. All datasets were tested by unpaired T test. * Significantly different from C57 by unpaired T test.

**Figure 4:**
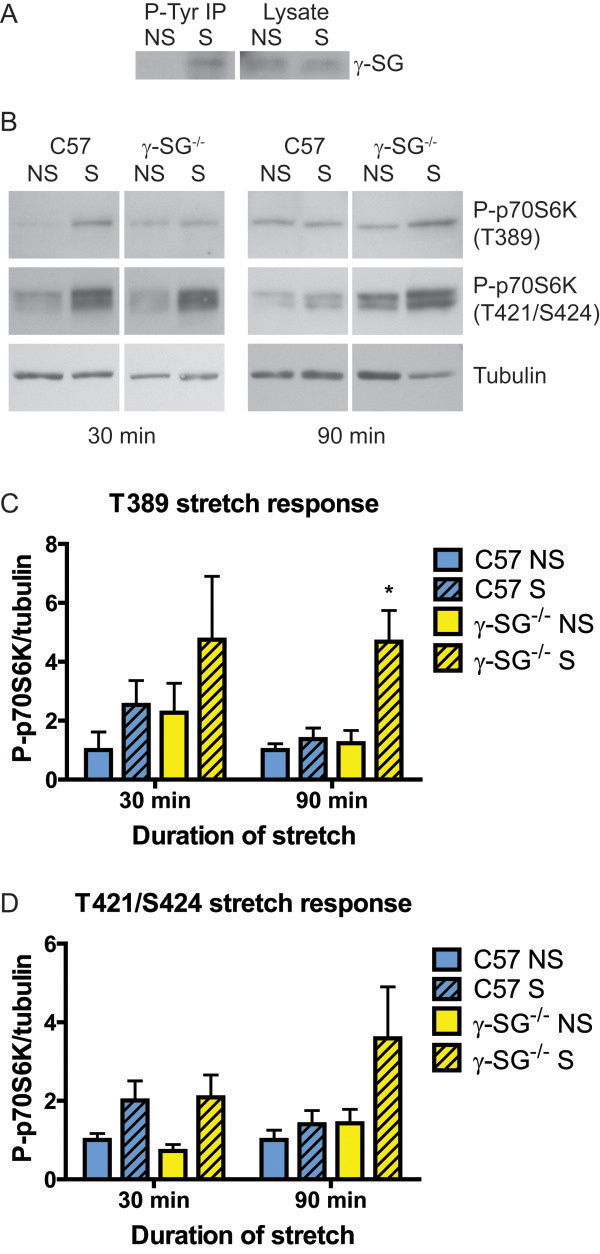
**Differential p70S6K stretch response in isolated C57 and γ-SG**^**-/- **^**muscles.** EDL muscles from C57 and γ-SG^-/-^ mice were maintained in oxygenated high glucose DMEM and subjected to passive stretching for 30 or 90 min. **(A)** Immunoblot of γ-SG following immunoprecipitation with anti-P-Tyr or lysate only, showing γ-SG phosphorylation in response to 30 min of stretch. **(B)** Representative immunoblots of P-p70S6K (T389 and T421/S424 sites) and tubulin. **(C, D)** Quantification of P-p70S6K T389 **(C)** and T421/S424 **(D)**, normalized to tubulin. Samples for each time point were run on separate immunoblots and normalized to C57 NS. n = 3-5 muscles per genotype, condition, and time point. Bars represent mean ± standard error. All datasets were tested by two-way ANOVA. Stretch was statistically significant for T421/S424 after 30 min of stretch; genotype, stretch, and the interaction between them were statistically significant T389 after 90 min of stretch, by two-way ANOVA. *Significantly different from C57 S at 90 min by two-way ANOVA with Tukey’s multiple comparisons test. NS, non-stretched; S, stretched.

**Figure 5:**
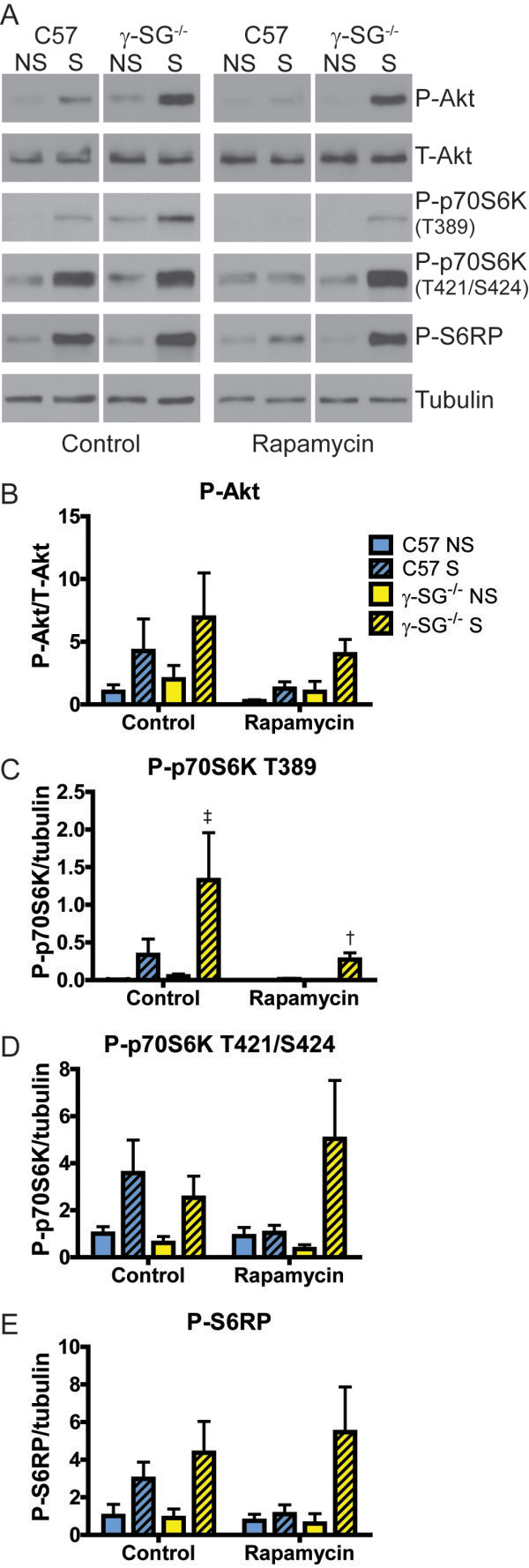
**Stretch response of p70S6K T389, but not S6RP, is rapamycin-sensitive in γ-SG**^**-/- **^**muscles.** EDL muscles from C57 and γ-SG^-/-^ mice were maintained in oxygenated high glucose DMEM supplemented with or without rapamycin and subjected to passive stretching for 90 min. **(A)** Representative immunoblots of P-Akt, T-Akt, P-p70S6K (T389 and T421/S424 sites), P-S6RP, and tubulin. Left panels DMEM alone; right panels DMEM + rapamycin. **(B-E)** Quantification of P-Akt **(B)**, P-p70S6K T389 **(C)**, P-p70S6K T421/S424 **(D)**, and P-S6RP **(E)**. Legend in B applies to all graphs. P-Akt was normalized to T-Akt; all other proteins were normalized to tubulin. n = 2-3 muscles per genotype and condition. Bars represent mean ± standard error. All datasets were tested by two-way ANOVA. For P-Akt in γ-SG^-/-^ muscles, stretch was significant. For P-p70S6K T389 in γ-SG^-/-^ muscles, stretch, rapamycin treatment, and the interaction between them were all significant. For P-S6RP in γ-SG^-/-^ muscles, stretch was significant. ‡Significantly different to NS γ-SG^-/-^ control and †significantly different to S γ-SG^-/-^ without rapamycin by two-way ANOVA with Tukey’s multiple comparisons test. NS, non-stretched; S, stretched.

Comparisons between non-stretched and stretched C2C12 cells (Figure 1) were done by unpaired T test. Comparisons between C57 and γ-SG^-/-^ primary cultures across time (Figure 2) were done by two-way ANOVA with Tukey’s multiple comparisons test. Comparisons between C57 and γ-SG^-/-^ muscles in calcium experiments (Figure 3) were done by unpaired T test. Comparisons between C57 and γ-SG^-/-^ muscles with and without stretch for each time point (Figure 4) or with and without rapamycin (Figure 5) were done by two-way ANOVA with Tukey’s multiple comparisons test.

## Results

### p70S6K, but not ERK1/2 or Akt responds to passive stretch *in vitro*

Studies using whole muscles from animal models of the dystrophies are made more complex by the presence of multiple cell types, as well as pathological processes such as fibrosis. Therefore, we initially investigated mechanotransduction signaling in passively stretched C2C12 myotubes. We found that passive stretching *in vitro* did not alter phosphorylation of ERK1 or Akt and that ERK2 and FAK phosphorylation decreased following stretch (Figure 1A,C-F,H-I). However, we found an increase in p70S6K phosphorylation at T389, which reflects activity, in response to passive stretching of C2C12 myotubes (Figure 1B,G). Therefore, this *in vitro* model reflected some, but not all, stretch responses observed in muscle *in vivo*[43,44], and highlighted p70S6K as a pathway of interest. The lack of Akt phosphorylation suggests that p70S6K phosphorylation occurred through an Akt-independent pathway, while the lack of FAK phosphorylation supports an integrin-independent mechanism.

### Differential p70S6K stretch response occurs in C57 and γ-SG^-/-^ primary cultures

Having established pathways of interest *in vitro* using the C2C12 cell line, we used primary myoblast cultures from C57 and γ-SG^-/-^ mice (Figure 2A) to investigate the changes in mechanotransduction signaling associated solely with the loss of the SG complex in myofibers. C57 and γ-SG^-/-^ cultures were stretched for 30 min as described above and lysates were harvested 1, 2, or 4 h after stretching ended, to allow observation of the signaling time course. Immunoblotting analysis showed that there was no difference in basal ERK1/2 phosphorylation between C57 and γ-SG^-/-^ myotubes and little change in ERK1/2 phosphorylation in response to stretch (Figure 2B,D,E). Therefore, similar to the C2C12 cells, primary cultures did not reflect the ERK1/2 phosphorylation responses found previously in C57 and γ-SG^-/-^ mice *in vivo*[10,11]. Neither P-Akt nor P-FAK displayed significant differences between C57 and γ-SG^-/-^ cultures (Figure 2B,F,G). Further, these proteins did not show any prolonged response to passive stretch in the primary cultures, consistent with the lack of an acute positive stretch response in C2C12 cells. Examination of p70S6K revealed a trend towards elevated T389 phosphorylation at baseline in γ-SG^-/-^ myotubes, compared to C57 myotubes. Activation of p70S6K in γ-SG^-/-^ myotubes upon stretch was not apparent (Figure 2B,C), but C57 myotubes displayed a trend for increased activation at 2 and 4 h after stretching (Figure 2B,C). Therefore, while the primary cultures reflect some of the responses found in C2C12 myotubes, the experimental system is too variable to draw firm conclusions regarding mechanical signaling pathways associated with γ-SG.

### Elevated P-p70S6K in γ-SG^-/-^ muscles is calcium independent

Our experiments in myotubes, together with our previous studies in isolated muscles [10,11], suggested that ERK1/2 phosphorylation changes require active contraction in addition to stretch, whereas p70S6K responds to stretch alone. To test this hypothesis *in vivo*, we extended our analysis to examine the response of p70S6K to passive stretch of isolated muscles. P-p70S6k was elevated approximately 1.7-fold in resting γ-SG^-/-^ EDL muscles incubated in normal oxygenated Ringer’s solution (Figure 3C; Normal). As in our previous studies, there was a 3- and 1.5-fold increase of P-ERK1 and P-ERK2, respectively, in resting γ-SG^-/-^ EDL muscles (Figure 3A-B; Normal). Because both of these pathways converge to phosphorylate S6RP, we compared the phosphorylation state of this protein in muscles from both genotypes. γ-SG^-/-^ EDL muscles exhibited an 8-fold increase in P-S6RP, which was consistent with the higher basal phosphorylation state of the upstream pathways (Figure 3D; Normal).

Heightened mechanosensitive signaling could arise through increased flux of ions across the sarcolemma, particularly Ca^2+^[17]. This could occur either through enhanced activity of channels, such as the TRP family of cation channels (reviewed in [28,29]), or via membrane ruptures. To determine the calcium dependence of the observed differences in p70S6K and ERK1/2 phosphorylation in γ-SG^-/-^ muscles, we incubated muscles in calcium-free Ringers solution, retaining the same ionic strength. Absence of extracellular Ca^2+^ did not alter the relative difference in P-p70S6K between γ-SG^-/-^ and C57 muscles (Figure 3C). However, the increased P-ERK1/2 and P-S6RP found in γ-SG^-/-^ muscles in normal Ringer’s solution was abrogated when there was no calcium in the bathing solution (Figure 3A,B,D). Thus, only p70S6K phosphorylation appeared to be calcium independent. Because intracellular calcium stores can also alter the intracellular calcium concentration, particularly during muscle activation, tetracaine was used to inhibit sarcoplasmic reticulum release of Ca^2+^ through the ryanodine receptors. Verification of this inhibition was established in a separate experiment, through measuring tetanic force generation by EDL muscles before and after addition of tetracaine. After 15 min incubation with tetracaine, force production was virtually eliminated (force was 297 mN prior to addition of tetracaine, 2.4 mN after 15 min incubation with tetracaine and not detectable after 20 min incubation with tetracaine). Again, relative P-p70S6K levels between the two muscle groups were not altered by tetracaine (Figure 3C). In contrast, blockade of SR Ca^2+^ release reduced P-ERK1 levels in γ-SG^-/-^ muscles relative to C57 muscles, even though there was no alteration in P-ERK2 (Figure 3A,B). Phosphorylation of S6RP remained elevated in γ-SG^-/-^ muscles in the presence of tetracaine, but to a lesser extent than in normal Ringer’s solution (Figure 3D). Taken together, both extracellular and intracellular calcium contribute to the heightened P-ERK1 levels in γ-SG^-/-^ muscles, whereas the increase in basal P-p70S6K in γ-SG^-/-^ muscles is not dependent on either extracellular or intracellular calcium.

### Differential p70S6K stretch response occurs in isolated C57 and γ-SG^-/-^ muscles

Having established that P-p70S6K changes at rest did not depend on calcium, we pursued the role of p70S6K in γ-SG-dependent mechanotransduction *in vivo*. A passive stretching protocol comprised of a 15% strain, 20 times per min, for 30 min in high-glucose DMEM was sufficient to cause increased γ-SG phosphorylation in the EDL, as is the case for eccentric contraction of the EDL (Figure 4A; [10]). We stretched C57 and γ-SG^-/-^ EDL muscles for either 30 or 90 min and immediately snap-froze them in liquid nitrogen. We then used immunoblotting to measure phosphorylation of p70S6K at T389, as above, and also at T421/S424 in the auto-inhibitory domain. Basal phosphorylation of p70S6K at T389 showed a trend to be increased in unstretched γ-SG^-/-^ muscles compared to C57 muscles (Figure 4B,C). However, basal phosphorylation at T421/S424 was not different between C57 and γ-SG^-/-^ muscles (Figure 4D). After 30 min of stretch, phosphorylation of p70S6K at T389 and T421/S424 was increased to a similar degree in stretched C57 and γ-SG^-/-^ EDL muscles, compared to non-stretched controls. However, after 90 min of stretch, phosphorylation at T389 and T421/S424 had decreased in stretched C57 muscles and was close to non-stretched levels. In contrast, phosphorylation at T389 was further increased, and T421/S424 phosphorylation remained elevated, in stretched γ-SG^-/-^ muscles, compared to non-stretched controls (Figure 4B-D). For T389 phosphorylation after 90 min of stretch, genotype, stretch, and the interaction between them were all statistically significant, by two-way ANOVA. T421/S424 showed a similar trend to T389; however, while the effect of stretch was statistically significant, the difference between genotypes was not. Thus, in contrast to primary cultures, γ-SG^-/-^ muscles exhibited heightened and prolonged activation of p70S6K in response to passive stretch, implying that γ-SG plays a role in p70S6K inactivation.

### Stretch response of p70S6K T389, but not S6RP, is rapamycin-sensitive in γ-SG^-/-^ muscles

Because mTOR is a key mediator of p70S6K activation, we examined the effect of the mTOR inhibitor rapamycin on stretch responses in isolated C57 and γ-SG^-/-^ muscles. EDL muscles were subjected to cyclic stretch for 90 min, as described above. Unlike in C2C12 cells and primary cultures, P-Akt showed a trend to increase on stretching, which was statistically significant in γ-SG^-/-^ muscles. As anticipated, P-Akt was unaffected by rapamycin (Figure 5A,B). Rapamycin treatment completely blocked the increase in p70S6K T389 phosphorylation after passive stretch of C57 muscles, consistent with previous studies [36,37]. In γ-SG^-/-^ muscles, rapamycin abrogated most p70S6K T389 phosphorylation, but residual phosphorylation remained in stretched muscles (Figure 5A,C). T421/S424 showed a trend to increase in response to stretch in both C57 and γ-SG^-/-^ muscles. Surprisingly, rapamycin blunted the p70S6K T421/S424 stretch response in C57 muscles, which is inconsistent with previous studies [36]. However, the T421/S424 response to stretch persisted in γ-SG^-/-^ muscles in the presence of rapamycin (Figure 5A,D). S6RP phosphorylation increased in response to stretch in both C57 and γ-SG^-/-^ muscles. Interestingly, while rapamycin blocked stretch-induced phosphorylation of S6RP in C57 muscles, phosphorylation in response to stretch was preserved in γ-SG^-/-^ muscles (Figure 5A,E). Taken together, these results suggest either that the level of active p70S6K remaining in γ-SG^-/-^ muscles is sufficient to phosphorylate S6RP regardless of rapamycin or that an alternate pathway bypasses p70S6K to phosphorylate S6RP in muscles lacking γ-SG.

## Discussion

Skeletal muscle has a remarkable ability to adapt to changes in workload. Almost all muscle properties can be modulated, such as muscle fiber size, contractile properties and metabolism. Changes in patterns of gene expression as well as shifts in the balance between protein synthesis and degradation are required to complete the adaptational response. Identification of major pathways that directly regulate gene expression and protein synthesis/degradation demonstrate that multiple inputs (mechanical, chemical, and metabolic) can converge on final common pathways for muscle growth and adaptation (reviewed in [45]). However, sorting out the contribution of the wide variety of inputs on muscle adaptation has been more difficult. In our previous work, we used an eccentric contraction protocol to investigate the dependence of ERK1/2 mechano-sensing on phosphorylation of γ-SG. However, this protocol alters multiple factors, including externally applied tension, internally generated tension and changes in extracellular and intracellular calcium fluxes, all of which potentially have effects on mechanosensitive signaling pathways. In the present study, we used a passive stretching protocol to isolate the effects of externally applied tension in the absence of active contraction, in order to examine the downstream signaling in more detail.

Passive stretching protocols can be performed in both cell cultures and whole muscle preparations, and the reductionist approach of utilizing cultures can eliminate some of the physiological complexities associated with intact or isolated muscles. As such, our initial experiments using C2C12 cells were key to identifying p70S6K as being activated in response to stretch, in contrast to the lack of response by ERK1/2, Akt, or FAK. Primary myotubes generated from C57 or γ-SG^-/-^ mice had the distinct advantage of efficient germline elimination of γ-SG combined with an *in vitro* culture system. Even though these experiments displayed trends towards differential activation of p70S6K after stretch, the inherent variability of the preparation impaired identification of signaling patterns that were dependent upon either stretch or γ-SG. Thus, we returned to isolated muscles from C57 and γ-SG^-/-^ mice to investigate γ-SG-dependent mechanotransduction pathways. Using this model, we observed a modest increase of P-p70S6K in γ-SG^-/-^ muscles at rest that was independent of intra- or extracellular calcium, and a prolonged activation of p70S6K following stretch. These results support a role for γ-SG in particular, or the SG complex in general, in mechanical signal transduction, where the loss of this protein leads to an increase in activation, and deficit in deactivation, or a combination of both. Given the dependence of our findings on the experimental platform utilized, future directions will include verification of the results in an even more intact system, such as *in situ* muscle preparations or whole animals.

An intriguing explanation for our *in vivo* results is that γ-SG is required for dephosphorylation and deactivation of p70S6K. There is considerable evidence that p70S6K is directly dephosphorylated by protein phosphatase 2A (PP2A), independently of mTOR [46-49]. The phosphatase PHLPP has also been shown to target p70S6K [50]. γ-SG may mediate the activation of these phosphatases in response to sustained mechanical stimulation. Alternatively, γ-SG may regulate pathways that deactivate p70S6K indirectly. For example, the phosphatase SHP-2 can cause mTOR-dependent dephosphorylation of p70S6K [51,52]. Further studies will be required to define the inactivation pathway disrupted by γ-SG loss.

Passive stretch eliminates the contribution of active contraction or SR calcium fluxes, but does not eliminate the effects of extracellular Ca^2+^ fluxes through mechanically sensitive channels. Further, as previously shown, passive stretch causes greater Ca^2+^ influx into myotubes lacking members of the SG complex [17], raising the possibility that the mechanical signal transduction pathways we evaluated previously may be modulated not only by the SG complex, but also by additional channels in the sarcolemma. To address this, we examined the contribution of calcium to the elevated p70S6K and ERK1/2 activity found in γ-SG^-/-^ muscles. We found that the elevation of P-ERK1/2 in the absence of γ-SG was dependent on both internal and external sources of calcium. In contrast, the difference in basal P-p70S6K between C57 and γ-SG^-/-^ muscles was not calcium dependent. This suggests that while ERK1/2 activation may lie downstream of the calcium misregulation that occurs in SG-deficient muscle, changes in p70S6K activation may be a more direct consequence of the absence of the SG complex. This is of great interest given that γ-SG has been shown to be important for mechanotransduction, but the downstream signaling pathways are uncharacterized [10,11]. Furthermore, p70S6K has been implicated in mechanotransduction in skeletal muscle, but the upstream initiation signals are not known [36,53-55]. However, it should be noted that SG-deficient muscle undergoes substantial degeneration and subsequent regeneration, which may also explain the elevated basal p70S6K, which is transiently increased during regeneration [56].

Our study found that the pattern of differential p70S6K phosphorylation in response to stretch in γ-SG^-/-^ muscles was similar both for phosphorylation of T389, which correlates with kinase activity, and for T421/S424, two of the four phosphorylation sites in the autoinhibitory domain. Phosphorylation of T389 is mTOR-dependent, while phosphorylation of the autoinhibitory domain is carried out by proline-directed kinases. Furthermore, it is thought that phosphorylation of the autoinhibitory domain is necessary for phosphorylation of T389 [37]. The correlation between phosphorylation of T421/S424 and T389 in our isolated muscle model therefore suggests that phosphorylation of the autoinhibitory domain was the rate-limiting step for p70S6K activation, an intriguing prospect given that the autoinhibitory domain may be targeted by ERK1/2 [57]. Therefore, a future hypothesis to test is that differential p70S6K activation is a downstream consequence of differential ERK1/2 activation in γ-SG^-/-^ muscle. This would implicate Ca^2+^ as an indirect modulator of p70S6K activity, since the increase in P-ERK1/2 in γ-SG^-/-^ muscle is dependent upon heightened Ca^2+^ flux. It is worth noting that recent work by others has shown that stretch-induced activation of mTOR and p70S6K at T389 is independent of ERK1/2 [54], which can regulate mTOR via tuberous sclerosis proteins 1 and 2 and Raptor [58-60]. However, this pathway is separate from the putative direct phosphorylation of the p70S6K T421/S424 autoinhibitory domain sites by ERK1/2.

Our experiments with rapamycin showed that, for both C57 and γ-SG^-/-^ muscles, phosphorylation of p70S6K at T389 is mTOR-dependent, consistent with previous studies [36,37]. The T421/S424 autoinhibitory domain sites were phosphorylated in response to stretch in both C57 and γ-SG^-/-^ muscles, which was different to our initial experiment in stretched isolated muscles, where the C57 response had diminished by 90 min of stretching. However, in the presence of rapamycin, this response was not present. This is surprising given that the T421/S424 sites are not thought to be targeted by mTOR. Previous studies have shown these sites to be rapamycin-insensitive [36], but recent evidence suggests a modest mTOR dependence [61]. In γ-SG^-/-^ muscles, rapamycin had no effect on T421/S424 phosphorylation. Further experiments are needed to fully understand the role of mTOR on phosphorylation of the p70S6K autoinhibitory domain in C57 and γ-SG^-/-^ skeletal muscle. Interestingly, the stretch-induced phosphorylation of S6RP was rapamycin sensitive in C57 muscles, but not in γ-SG^-/-^ muscles. This suggests that an alternative pathway can bring about S6RP phosphorylation in γ-SG^-/-^ muscles when p70S6K is not activated. One possibility is that S6RP is phosphorylated by p90 ribosomal S6 kinase, which is activated by ERK; this is consistent with the increase in basal ERK1/2 in γ-SG^-/-^ muscles, and the over-response of ERK2 on mechanical stimulation by eccentric contraction [10].

**Figure 6:**
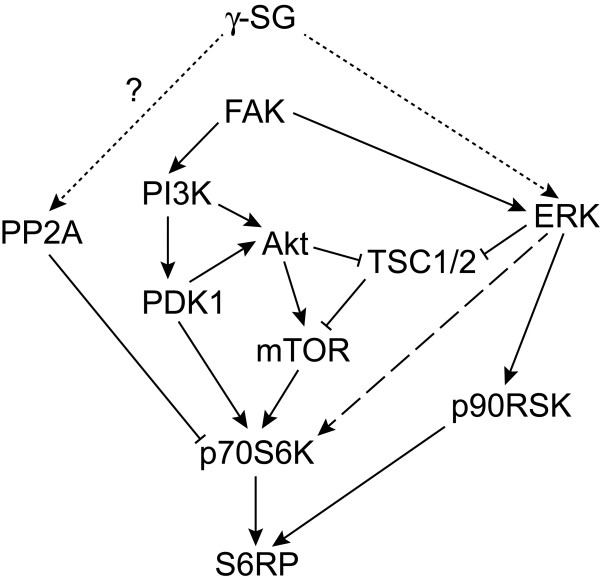
**Relevant signaling pathways and relationship to γ-SG.** Schematic of signaling pathways measured or discussed in this manuscript. Dotted lines indicate possible relationships to γ-SG. Arrowheads indicate an activating relationship, while blunt ends indicate a repressing relationship. Dashed line indicates priming, rather than full activation.

We did not observe a strong Akt response to passive stretch, or any difference between C57 and γSG^-/-^, implying that mTOR and/or p70S6K were being activated through Akt-independent pathways. This is consistent with previous studies showing that Akt does not respond to mechanical stimulation in skeletal muscle, and that p70S6K phosphorylation in response to stretch is independent of PI3K [62]. We also did not see increased phosphorylation of FAK in response to passive stretch in C2C12 cells or primary myotubes. Although integrins can participate in mechanotransduction, it appears that our cyclic passive stretch protocols did not cause integrin activation. Further studies will be needed to elucidate the details of crosstalk between SG-dependent and integrin-dependent signaling pathways, as well as the role of calcium in these signaling cascades.Based on our findings, we position γSG as a mechanosensor, schematized in Figure 6, that is important for transient ERK1/2 activation during active contractions, as well as modulation of p70S6K activation during passive stretch. Because passive stretch does not appear to increase P-FAK or P-Akt, γSG is likely to regulate p70S6K through other pathways. These may include regulation of ERK1/2, which can promote p70S6K activation indirectly via mTOR or directly by phosphorylation of the autoinhibitory domain, and/or phosphatases such as PP2A that dephosphorylate p70S6K. Loss of γSG uncouples the response to stretch, which may contribute to muscle pathology.

The stability of the SG complex is directly affected in several LGMDs and in DMD, and a significant part of the pathology in these diseases appears to be inappropriate load sensing. The first step in therapeutic development is identifying and understanding the target, but little is currently known about the role of the SG complex in load sensing. Therefore, understanding the functions that are disrupted and the pathways that are involved in mechanotransduction involving the SG complex will help in the design of therapies for LGMDs and DMD. While restoration of a completely normal SG complex either through gene correction or protein replacement would also normalize mechanical signal transduction, this may not be possible for all mutations responsible for DMD and LGMD. It is known that localization of the SG complex is not the sole criterion for appropriate signaling [11]. Hence, other proteins may be necessary to correct signaling even when the complex is restored, and downstream pathways may emerge as more feasible therapeutic targets. We do not know whether the enhanced basal and stretch-responsive activation of p70S6K in γ-SG^-/-^ muscle contributes to pathology or compensates for it. Likewise, it is not clear whether inhibition of p70S6K would have a beneficial or a detrimental effect in dystrophic muscle. Muscle specific gene targeting of mTORC1 components induces myopathy [63,64] and overexpression of integrin α7 can improve the dystrophic phenotype through increased survival signaling via p70S6K [65], suggesting that p70S6K inhibition would not be advantageous. However, treatment of *mdx* mice with the mTOR inhibitor rapamycin improves the dystrophic phenotype [66]. It is also interesting to note that p70S6K is inhibited by glucocorticoids, which are used in the treatment of DMD and LGMD [67].

Our results begin to provide mechanistic insight into how mechanical signaling is disrupted and altered in the absence of γ-SG. In addition to increasing our understanding of the normal function of the SG complex, there is potential to provide more refined targets that could be beneficial to patients either in isolation or in combination with other therapeutic approaches.

## Conclusions

We have identified p70S6K as part of a novel SG-dependent mechanosensitive signaling pathway in skeletal muscle. Our results suggest that γ-SG is required for the inactivation of p70S6K following its activation in response to mechanical stimulation. These studies provide new insights into the normal function of the SG complex, and the mechanisms by which its deficiency in some forms of muscular dystrophy may contribute to pathology.

## Competing interests

The authors declare that they have no competing interests.

## Authors’ contributions

CM carried out the calcium dependence experiments and the stretching experiments in isolated muscles, and drafted the manuscript. AP carried out the passive stretching experiments in primary myotube cultures. JS carried out the passive stretching experiments in C2C12 myotubes, the immunoprecipitation of phosphorylated γ-SG, and the immunoblotting for rapamycin sensitivity experiments. BK participated in the passive stretching experiments in primary cultures. EJM developed the apparatus used for stretching of myotube cultures. ERB conceived of the study, participated in its design, coordination, and interpretation of the results, and helped draft the manuscript. All authors read and approved the final manuscript.

## References

[B1] Ervasti JM Sonnemann KJ Biology of the striated muscle dystrophin-glycoprotein complex Int Rev Cytol 2008 265 191 225 18275889 10.1016/S0074-7696(07)65005-0

[B2] Emery AE Muntoni F Duchenne Muscular Dystrophy 2003 Oxford: Oxford University Press

[B3] Engel AG Franzini-Armstrong C Myology: Basic and Clinical 2004 3 New York: McGraw-Hill, Inc

[B4] Durbeej M Campbell KP Muscular dystrophies involving the dystrophin-glycoprotein complex: an overview of current mouse models Curr Opin Genet Dev 2002 12 349 361 12076680 10.1016/s0959-437x(02)00309-x

[B5] Blake DJ Weir A Newey SE Davies KE Function and genetics of dystrophin and dystrophin-related proteins in muscle Physiol Rev 2002 82 291 329 11917091 10.1152/physrev.00028.2001

[B6] Davies KE Nowak KJ Molecular mechanisms of muscular dystrophies: old and new players Nat Rev 2006 7 762 773 10.1038/nrm2024 16971897

[B7] Hack AA Cordier L Shoturma DI Lam MY Sweeney HL McNally EM Muscle degeneration without mechanical injury in sarcoglycan deficiency Proc Natl Acad Sci U S A 1999 96 10723 10728 10485893 10.1073/pnas.96.19.10723 PMC17950

[B8] Petrof BJ Shrager JB Stedman HH Kelly AM Sweeney HL Dystrophin protects the sarcolemma from stresses developed during muscle contraction Proc Natl Acad Sci 1993 90 3710 3714 8475120 10.1073/pnas.90.8.3710 PMC46371

[B9] Hack AA Ly CT Jiang F Clendenin CJ Sigrist KS Wollmann RL McNally EM Gamma-sarcoglycan deficiency leads to muscle membrane defects and apoptosis independent of dystrophin J Cell Biol 1998 142 1279 1287 9732288 10.1083/jcb.142.5.1279 PMC2149352

[B10] Barton ER Impact of sarcoglycan complex on mechanical signal transduction in murine skeletal muscle Am J Physiol Cell Physiol 2006 290 C411 C419 16162659 10.1152/ajpcell.00192.2005

[B11] Barton ER Restoration of gamma-sarcoglycan localization and mechanical signal transduction are independent in murine skeletal muscle J Biol Chem 2010 285 17263 17270 20371873 10.1074/jbc.M109.063990 PMC2878052

[B12] Benavides Damm T Egli M Calcium’s role in mechanotransduction during muscle development Cell Physiol Biochem 2014 33 249 272 24525559 10.1159/000356667

[B13] Burkholder TJ Mechanotransduction in skeletal muscle Front Biosci 2007 12 174 191 17127292 10.2741/2057 PMC2043154

[B14] Andersson D Meli A Reiken S Betzenhauser M Umanskaya A Shiomi T D’Armiento J Marks A Leaky ryanodine receptors in beta-sarcoglycan deficient mice: a potential common defect in muscular dystrophy Skeletal Muscle 2012 2 9 22640601 10.1186/2044-5040-2-9 PMC3605002

[B15] Iwata Y Katanosaka Y Shijun Z Kobayashi Y Hanada H Shigekawa M Wakabayashi S Protective effects of Ca2+ handling drugs against abnormal Ca2+ homeostasis and cell damage in myopathic skeletal muscle cells Biochem Pharmacol 2005 70 740 751 16009351 10.1016/j.bcp.2005.05.034

[B16] Nakamura TY Iwata Y Sampaolesi M Hanada H Saito N Artman M Coetzee WA Shigekawa M Stretch-activated cation channels in skeletal muscle myotubes from sarcoglycan-deficient hamsters Am J Physiol Cell Physiol 2001 281 C690 C699 11443068 10.1152/ajpcell.2001.281.2.C690

[B17] Sampaolesi M Yoshida T Iwata Y Hanada H Shigekawa M Stretch-induced cell damage in sarcoglycan-deficient myotubes Pflugers Arch 2001 442 161 170 11417209 10.1007/s004240100516

[B18] Solares-Perez A Alvarez R Crosbie RH Vega-Moreno J Medina-Monares J Estrada FJ Ortega A Coral-Vazquez R Altered calcium pump and secondary deficiency of gamma-sarcoglycan and microspan in sarcoplasmic reticulum membranes isolated from delta-sarcoglycan knockout mice Cell Calcium 2010 48 28 36 20638123 10.1016/j.ceca.2010.06.003 PMC4859437

[B19] Solares-Perez A Sanchez JA Zentella-Dehesa A Garcia MC Coral-Vazquez RM Intracellular Ca2+ transients in delta-sarcoglycan knockout mouse skeletal muscle Biochim Biophys Acta 2010 1800 373 379 19931597 10.1016/j.bbagen.2009.11.011

[B20] Hassoni C Calcium homeostasis and ultrastructural studies in a patient with limb girdle muscular dystrophy type 2C Neuropathol Appl Neurobiol 1999 25 244 253 10417666 10.1046/j.1365-2990.1999.00169.x

[B21] Fraysse B Nagi SM Boher B Ragot H Lainé J Salmon A Fiszman MY Toussaint M Fromes Y Ca2+ overload and mitochondrial permeability transition pore activation in living δ-sarcoglycan-deficient cardiomyocytes Am J Physiol Cell Physiol 2010 299 C706 C713 20592245 10.1152/ajpcell.00545.2009

[B22] Lipskaia L Pinet C Fromes Y Hatem S Cantaloube I Coulombe A Lompré A-M Mutation of δ-sarcoglycan is associated with Ca2 + -dependent vascular remodeling in the Syrian hamster Am J Pathol 2007 171 162 171 17591963 10.2353/ajpath.2007.070054 PMC1941595

[B23] Kumar A Khandelwal N Malya R Reid MB Boriek AM Loss of dystrophin causes aberrant mechanotransduction in skeletal muscle fibers FASEB J 2004 18 102 113 14718391 10.1096/fj.03-0453com

[B24] Iwata Y Katanosaka Y Arai Y Komamura K Miyatake K Shigekawa M A novel mechanism of myocyte degeneration involving the Ca2 + -permeable growth factor–regulated channel J Cell Biol 2003 161 957 967 12796481 10.1083/jcb.200301101 PMC2172975

[B25] Iwata Y Katanosaka Y Arai Y Shigekawa M Wakabayashi S Dominant-negative inhibition of Ca2+ influx via TRPV2 ameliorates muscular dystrophy in animal models Hum Mol Genet 2009 18 824 834 19050039 10.1093/hmg/ddn408

[B26] Millay DP Goonasekera SA Sargent MA Maillet M Aronow BJ Molkentin JD Calcium influx is sufficient to induce muscular dystrophy through a TRPC-dependent mechanism Proc Natl Acad Sci U S A 2009 106 19023 19028 19864620 10.1073/pnas.0906591106 PMC2776441

[B27] Goonasekera SA Davis J Kwong JQ Accornero F Wei-LaPierre L Sargent MA Dirksen RT Molkentin JD Enhanced Ca2+ influx from STIM1–Orai1 induces muscle pathology in mouse models of muscular dystrophy Hum Mol Genet 2014 [Epub ahead of print] 10.1093/hmg/ddu079 PMC4065147 24556214

[B28] Brinkmeier H TRP channels in skeletal muscle: gene expression, function and implications for disease Adv Exp Med Biol 2011 704 749 758 21290325 10.1007/978-94-007-0265-3_39

[B29] Gailly P TRP channels in normal and dystrophic skeletal muscle Curr Opin Pharmacol 2012 12 326 334 22349418 10.1016/j.coph.2012.01.018

[B30] Araishi K Sasaoka T Imamura M Noguchi S Hama H Wakabayashi E Yoshida M Hori T Ozawa E Loss of the sarcoglycan complex and sarcospan leads to muscular dystrophy in β-sarcoglycan-deficient mice Hum Mol Genet 1999 8 1589 1598 10441321 10.1093/hmg/8.9.1589

[B31] Duclos F Straub V Moore SA Venzke DP Hrstka RF Crosbie RH Durbeej M Lebakken CS Ettinger AJ van der Meulen J Holt KH Lim LE Sanes JR Davidson BL Faulkner JA Williamson R Campbell KP Progressive muscular dystrophy in α-sarcoglycan-deficient mice J Cell Biol 1998 142 1461 1471 9744877 10.1083/jcb.142.6.1461 PMC2141773

[B32] Goonasekera SA Lam CK Millay DP Sargent MA Hajjar RJ Kranias EG Molkentin JD Mitigation of muscular dystrophy in mice by SERCA overexpression in skeletal muscle J Clin Invest 2011 121 1044 1052 21285509 10.1172/JCI43844 PMC3049367

[B33] Morine KJ Sleeper MM Barton ER Sweeney HL Overexpression of SERCA1a in the mdx diaphragm reduces susceptibility to contraction-induced damage Hum Gene Ther 2010 21 1735 1739 20540606 10.1089/hum.2010.077 PMC2999573

[B34] Parsons SA Millay DP Sargent MA Naya FJ McNally EM Sweeney HL Molkentin JD Genetic disruption of calcineurin improves skeletal muscle pathology and cardiac disease in a mouse model of limb-girdle muscular dystrophy J Biol Chem 2007 282 10068 10078 17289669 10.1074/jbc.M609368200 PMC2644416

[B35] Fenton TR Gout IT Functions and regulation of the 70 kDa ribosomal S6 kinases Int J Biochem Cell Biol 2011 43 47 59 20932932 10.1016/j.biocel.2010.09.018

[B36] Hornberger TA Stuppard R Conley KE Fedele MJ Fiorotto ML Chin ER Esser KA Mechanical stimuli regulate rapamycin-sensitive signalling by a phosphoinositide 3-kinase-, protein kinase B- and growth factor-independent mechanism Biochem J 2004 380 795 804 15030312 10.1042/BJ20040274 PMC1224227

[B37] Dufner A Thomas G Ribosomal S6 kinase signaling and the control of translation Exp Cell Res 1999 253 100 109 10579915 10.1006/excr.1999.4683

[B38] Pearson RB Dennis PB Han JW Williamson NA Kozma SC Wettenhall RE Thomas G The principal target of rapamycin-induced p70s6k inactivation is a novel phosphorylation site within a conserved hydrophobic domain EMBO J 1995 14 5279 5287 7489717 10.1002/j.1460-2075.1995.tb00212.x PMC394637

[B39] Nielsen PJ Manchester KL Towbin H Gordon J Thomas G The phosphorylation of ribosomal protein S6 in rat tissues following cycloheximide injection, in diabetes, and after denervation of diaphragm. A simple immunological determination of the extent of S6 phosphorylation on protein blots J Biol Chem 1982 257 12316 12321 6749858

[B40] He Y Macarak EJ Korostoff JM Howard PS Compression and tension: differential effects on matrix accumulation by periodontal ligament fibroblasts in vitro Connect Tissue Res 2004 45 28 39 15203938 10.1080/03008200490278124

[B41] Barton ER Park S James JK Makarewich CA Philippou A Eletto D Lei H Brisson B Ostrovsky O Li Z Argon Y Deletion of muscle GRP94 impairs both muscle and body growth by inhibiting local IGF production FASEB J 2012 26 3691 3702 22649033 10.1096/fj.11-203026 PMC3425820

[B42] Moorwood C Liu M Tian Z Barton ER Isometric and eccentric force generation assessment of skeletal muscles isolated from murine models of muscular dystrophies J Vis Exp 2013 71 e50036 23407283 10.3791/50036 PMC3596910

[B43] Hornberger TA Mateja RD Chin ER Andrews JL Esser KA Aging does not alter the mechanosensitivity of the p38, p70S6k, and JNK2 signaling pathways in skeletal muscle J Appl Physiol (1985) 2005 98 1562 1566 15361519 10.1152/japplphysiol.00870.2004

[B44] Martineau LC Gardiner PF Insight into skeletal muscle mechanotransduction: MAPK activation is quantitatively related to tension J Appl Physiol (1985) 2001 91 693 702 11457783 10.1152/jappl.2001.91.2.693

[B45] Glass DJ Signalling pathways that mediate skeletal muscle hypertrophy and atrophy Nat Cell Biol 2003 5 87 90 12563267 10.1038/ncb0203-87

[B46] Hahn K Miranda M Francis VA Vendrell J Zorzano A Teleman AA PP2A regulatory subunit PP2A-B’ counteracts S6K phosphorylation Cell Metab 2010 11 438 444 20444422 10.1016/j.cmet.2010.03.015

[B47] Peterson RT Desai BN Hardwick JS Schreiber SL Protein phosphatase 2A interacts with the 70-kDa S6 kinase and is activated by inhibition of FKBP12-rapamycinassociated protein Proc Natl Acad Sci U S A 1999 96 4438 4442 10200280 10.1073/pnas.96.8.4438 PMC16350

[B48] Petritsch C Beug H Balmain A Oft M TGF-beta inhibits p70 S6 kinase via protein phosphatase 2A to induce G(1) arrest Genes Dev 2000 14 3093 3101 11124802 10.1101/gad.854200 PMC317138

[B49] Cho DH Choi YJ Jo SA Ryou J Kim JY Chung J Jo I Troglitazone acutely inhibits protein synthesis in endothelial cells via a novel mechanism involving protein phosphatase 2A-dependent p70 S6 kinase inhibition Am J Physiol Cell Physiol 2006 291 C317 C326 16825603 10.1152/ajpcell.00491.2005

[B50] Liu J Stevens PD Li X Schmidt MD Gao T PHLPP-mediated dephosphorylation of S6K1 inhibits protein translation and cell growth Mol Cell Biol 2011 31 4917 4927 21986499 10.1128/MCB.05799-11 PMC3233022

[B51] Ketroussi F Giuliani M Bahri R Azzarone B Charpentier B Durrbach A Lymphocyte cell-cycle inhibition by HLA-G is mediated by phosphatase SHP-2 and acts on the mTOR pathway PLoS One 2011 6 e22776 21887223 10.1371/journal.pone.0022776 PMC3160837

[B52] Zito CI Qin H Blenis J Bennett AM SHP-2 regulates cell growth by controlling the mTOR/S6 kinase 1 pathway J Biol Chem 2007 282 6946 6953 17229738 10.1074/jbc.M608338200

[B53] Hornberger TA Chu WK Mak YW Hsiung JW Huang SA Chien S The role of phospholipase D and phosphatidic acid in the mechanical activation of mTOR signaling in skeletal muscle Proc Natl Acad Sci U S A 2006 103 4741 4746 16537399 10.1073/pnas.0600678103 PMC1450240

[B54] You JS Frey JW Hornberger TA Mechanical stimulation induces mTOR signaling via an ERK-independent mechanism: implications for a direct activation of mTOR by phosphatidic acid PLoS One 2012 7 e47258 23077579 10.1371/journal.pone.0047258 PMC3471816

[B55] You JS Lincoln HC Kim CR Frey JW Goodman CA Zhong XP Hornberger TA The role of diacylglycerol kinase zeta and phosphatidic acid in the mechanical activation of mammalian target of rapamycin (mTOR) signaling and skeletal muscle hypertrophy J Biol Chem 2014 289 1551 1563 24302719 10.1074/jbc.M113.531392 PMC3894336

[B56] Richard-Bulteau H Serrurier B Crassous B Banzet S Peinnequin A Bigard X Koulmann N Recovery of skeletal muscle mass after extensive injury: positive effects of increased contractile activity Am J Physiol Cell Physiol 2008 294 C467 C476 18077604 10.1152/ajpcell.00355.2007

[B57] Mukhopadhyay NK Price DJ Kyriakis JM Pelech S Sanghera J Avruch J An array of insulin-activated, proline-directed serine/threonine protein kinases phosphorylate the p70 S6 kinase J Biol Chem 1992 267 3325 3335 1737788

[B58] Carriere A Romeo Y Acosta-Jaquez HA Moreau J Bonneil E Thibault P Fingar DC Roux PP ERK1/2 Phosphorylate Raptor to Promote Ras-dependent Activation of mTOR Complex 1 (mTORC1) J Biol Chem 2011 286 567 577 21071439 10.1074/jbc.M110.159046 PMC3013016

[B59] Ma L Chen Z Erdjument-Bromage H Tempst P Pandolfi PP Phosphorylation and Functional Inactivation of TSC2 by Erk: Implications for Tuberous Sclerosisand Cancer Pathogenesis Cell 2005 121 179 193 15851026 10.1016/j.cell.2005.02.031

[B60] Roux PP Ballif BA Anjum R Gygi SP Blenis J Tumor-promoting phorbol esters and activated Ras inactivate the tuberous sclerosis tumor suppressor complex via p90 ribosomal S6 kinase Proc Natl Acad Sci U S A 2004 101 13489 13494 15342917 10.1073/pnas.0405659101 PMC518784

[B61] Axelrod MJ Gordon V Mendez RE Leimgruber SS Conaway MR Sharlow ER Jameson MJ Gioeli DG Weber MJ p70S6 kinase is a critical node that integrates HER-family and PI3 kinase signaling networks Cell Signal 2014 26 1627 1635 24662264 10.1016/j.cellsig.2014.03.013 PMC4091927

[B62] Hornberger TA Chien S Mechanical stimuli and nutrients regulate rapamycin-sensitive signaling through distinct mechanisms in skeletal muscle J Cell Biochem 2006 97 1207 1216 16315321 10.1002/jcb.20671

[B63] Bentzinger CF Romanino K Cloëtta D Lin S Mascarenhas JB Oliveri F Xia J Casanova E Costa CF Brink M Zorzato F Hall MN Ruegg MA Skeletal muscle-specific ablation of raptor, but not of rictor, causes metabolic changes and results in muscle dystrophy Cell Metab 2008 8 411 424 19046572 10.1016/j.cmet.2008.10.002

[B64] Risson V Mazelin L Roceri M Sanchez H Moncollin V Corneloup C Richard-Bulteau H Vignaud A Baas D Defour A Freyssenet D Tanti JF Le-Marchand-Brustel Y Ferrier B Conjard-Duplany Romanino K Bauche S Hantai D Mueller M Kozma SC Thomas G Ruegg MA Ferry A Pende M Bigard X Koulmann N Schaeffer L Gangloff YG Muscle inactivation of mTOR causes metabolic and dystrophin defects leading to severe myopathy J Cell Biol 2009 187 859 874 20008564 10.1083/jcb.200903131 PMC2806319

[B65] Boppart MD Burkin DJ Kaufman SJ Activation of AKT signaling promotes cell growth and survival in α7β1 integrin-mediated alleviation of muscular dystrophy Biochim Biophys Acta Mol Basis Dis 2011 1812 439 446 10.1016/j.bbadis.2011.01.002 PMC3046458 21216283

[B66] Eghtesad S Jhunjhunwala S Little SR Clemens PR Effect of rapamycin on immunity induced by vector-mediated dystrophin expression in mdx skeletal muscle Sci Rep 2012 2 399 22570764 10.1038/srep00399 PMC3347316

[B67] Shah OJ Kimball SR Jefferson LS Glucocorticoids abate p70S6k and eIF4E function in L6 skeletal myoblasts Am J Physiol Endocrinol Metabol 2000 279 E74 E82 10.1152/ajpendo.2000.279.1.E74 10893325

